# Using a Robot to Treat Non-specific Low Back Pain: Results From a Two-Arm, Single-Blinded, Randomized Controlled Trial

**DOI:** 10.3389/fnbot.2021.715632

**Published:** 2021-09-14

**Authors:** Honorio Marín-Méndez, Patricia Marín-Novoa, Silvia Jiménez-Marín, Itziar Isidoro-Garijo, Mercedes Ramos-Martínez, Miriam Bobadilla, Eduardo Mirpuri, Alfredo Martínez

**Affiliations:** ^1^Rehabilitation Service, High Resolution Center San Millán, Servicio Riojano de Salud, Logroño, Spain; ^2^Oncology Area, Center for Biomedical Research of La Rioja (CIBIR), Logroño, Spain

**Keywords:** low back pain, robot, therapeutic massage, body mass index, overweight, perceived pain

## Abstract

Non-specific low back pain (NSLBP) affects many people and represents a high cost for health care. Manual pressure release of myofascial trigger points is used to treat NSLBP and is very effective but difficult to standardize since it is provided by different therapists, which also suffer musculoskeletal complications from this highly repetitive activity. A robot designed for this purpose may help in reducing these problems. Here, we present data from a two-arm, single-blinded, randomized controlled clinical trial evaluating the efficiency of a therapeutic massage robot (ADAMO) in reducing NSLBP (clinicaltrials.gov, registration number: NCT04882748). Forty-four patients were randomly distributed into the two arms of the study (robot vs. control). A physician filled the Oswestry disability index (ODI) before starting the treatment and at the end of it, in a blind fashion. In addition, patients filled a visual analogue scale (VAS) after each of the 10 treatment sessions. The ODI and the VAS were analyzed as the primary and secondary outcome measures. Both treatments (robot and control) resulted in a significantly lower ODI (*p* < 0.05). On the other hand, robot-treated patients significantly reduced their VAS levels (*p* = 0.0001) whereas control treatment did not reach statistical significance. Patients of both sexes obtained similar benefits from either treatment. Overweight patients (body mass index ≥ 25kg/m^2^) in the robot arm benefited more from the treatment (*p* = 0.008) than patients with normal weight. In conclusion, the ADAMO robot is, at least, as efficient as regular treatment in reducing low back pain, and may be more beneficial for specific patients, such as those with excessive weight.

## Introduction

Low back pain is defined as a musculoskeletal syndrome, or group of symptoms, whose main characteristic is the pain, which is focalized in the lumbar area of the spine. The diagnosis is rather easy since symptoms are very evident. When this pain cannot be attributed to a known cause (traumatism, systemic diseases, nerve root compression, etc), it is called non-specific low back pain (NSLBP) (Maher et al., 2017), which may represent 90–95% of all cases of back pain (Bardin et al., 2017).

NSLBP cannot be considered a benign pathology. On the contrary, it is responsible for a high index of work absenteeism and early retirement (Ekman et al., 2005; Hoy et al., 2014). This syndrome affects 70–80% of the population of developed countries at some stage during their lifetime, representing the main cause of motility restriction, long-term incapacity, and reduction in the quality of life. In Europe, it has an associated cost of between 1.7 and 2.1% of the gross domestic product (Lambeek et al., 2011), while in the US it costs about $ 100 billion a year (Dieleman et al., 2016).

The treatment for this pathology has been collected in several clinical practice guidelines, with little differences among them (Oliveira et al., 2018). All of them recommend: (i) maintaining physical activity as far as the pain allows; (ii) pharmacological treatment (analgesics, non-steroidal anti-inflammatories, muscle relaxants); and (iii) non-pharmacological measures (local heat, cognitive-behavior therapy, spinal manipulation, rehabilitation programs). Rehabilitation therapy, including different exercises, such as stretches, back workshops, and aquatic exercises, among others, provides excellent results in managing chronic back pain (Searle et al., 2015). In addition, manual pressure release of myofascial trigger points constitutes the most common practice to treat back pain and is very effective in the short term, although it may not address the underlying causes (Dayanir et al., 2020). These trigger points are hyperirritable zones located in a taut band of skeletal muscle that generate pain with compression, distension, overload, or contraction of the tissue, which usually responds with a referred pain (Moraska et al., 2017).

Pain evaluation is a fundamental requisite in the outcome assessment of any pain intervention. It is well-known that psychological and psychosocial factors may substantially influence pain perception, so different scales have been developed to measure the intensity of perceived pain in patients. The most extended ones are the visual analogue scale (VAS) and the Oswestry disability index (ODI) (Haefeli and Elfering, 2006; Mehra et al., 2008).

The main problem in measuring and reporting manual massage practices is that the massage is applied by different therapists, with different strength and intensity, which may vary from session to session (Farber and Wieland, 2016). The use of massaging robotic devices should solve all these problems and several prototypes have been proposed (Wang et al., 2018; Li et al., 2020).

**Figure 1:**
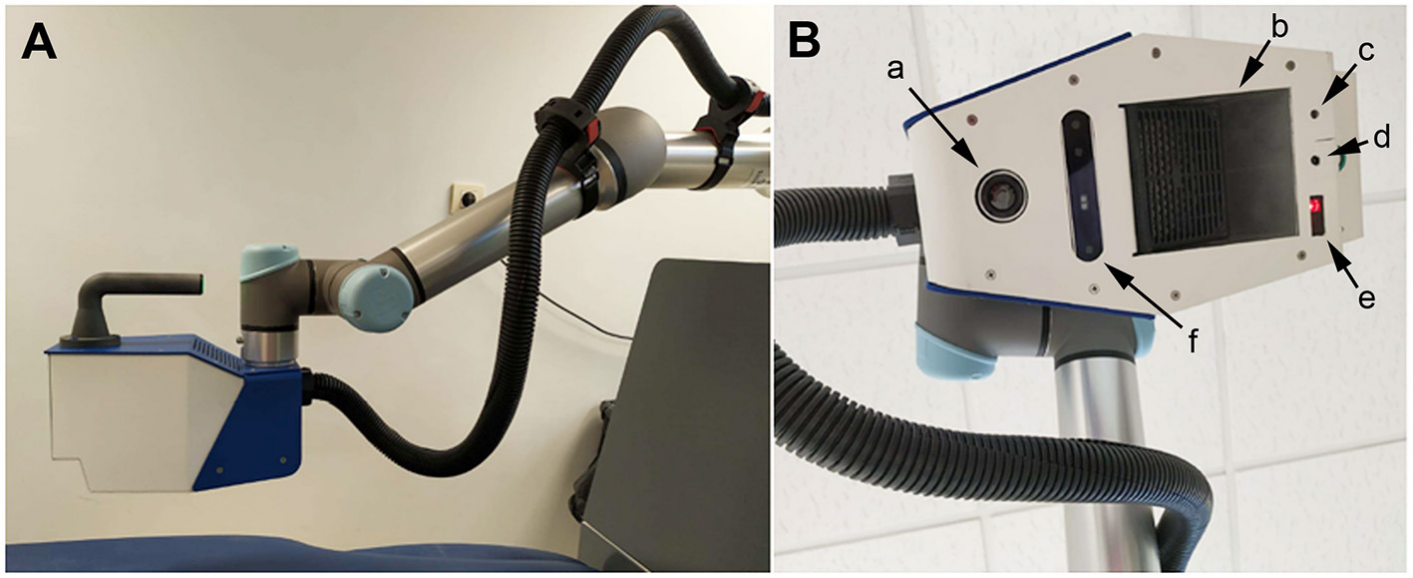
Representative photographs of the ADAMO device showing the robotic arm with the terminal handpiece **(A)**, and detail of the handpiece **(B)** indicating the location of the thermal camera **(a)**, the air heater **(b)**, the cross selection laser **(c)**, the air nozzle **(d)**, the distance sensor **(e)**, and the 3D camera **(f)**. The black tube carries compressed air.

In this study, we will test the efficiency of the new ADAMO robot system (https://adamorobot.com/), produced and distributed by Future Sense, Inc (Spain). ADAMO bases its operation on a computer program that controls the manipulator robot, which, by means of cameras installed at the end of its arm, must find in each session the points of treatment in the patient previously defined by the health professional and apply the necessary pressure. This pressure is generated by means of a compressed air nozzle integrated in a handpiece installed at the end of the robot arm (Figure 1).

The objective of our study was to measure the efficiency of adding the ADAMO robot to the current protocol of non-pharmacological measures in reducing NSLBP.

## Materials and Methods

This was a two-arm, single-blinded, randomized controlled trial. The study was approved by the local ethics committee (Comité de Ética de Investigación con Medicamentos de La Rioja, CEImLAR, protocol P.S 7) and registered at clinicaltrials.gov (registration number: NCT04882748). The study follows all tenets of the Declaration of Helsinki and was conducted at the Rehabilitation Service, High Resolution Center San Millán, in Logroño (Spain) between October 2020 and February 2021.

### Subjects

Patients of both sexes that arrived to the Rehabilitation Service seeking treatment for NSLBP were included in the trial if they were suffering from NSLBP, had between 18 and 60 years of age, and they signed the informed consent form. Participants were excluded if they fulfilled any of the following criteria: pregnancy, impossibility of staying in a prone position, previous pathologies (spinal surgery, cancer, rheumatic diseases, cardiopaties, respiratory compromise, etc), allergies and/or skin affectations.

Sample size was calculated based on data published by Patti et al. (2016). In that study, the pain of patients suffering from NSLBP was measured with the ODI. On their first visit, patients exhibited a pain rate of 13.7 ± 5.0, which after several sessions of Pilates exercises became 6.5 ± 4.0. Power was set as 80% for an alpha of 5% and attrition of 20%, resulting in 22 patients per study arm, in order to reach a significant relief at the end of treatment.

### Intervention

**Figure 2:**
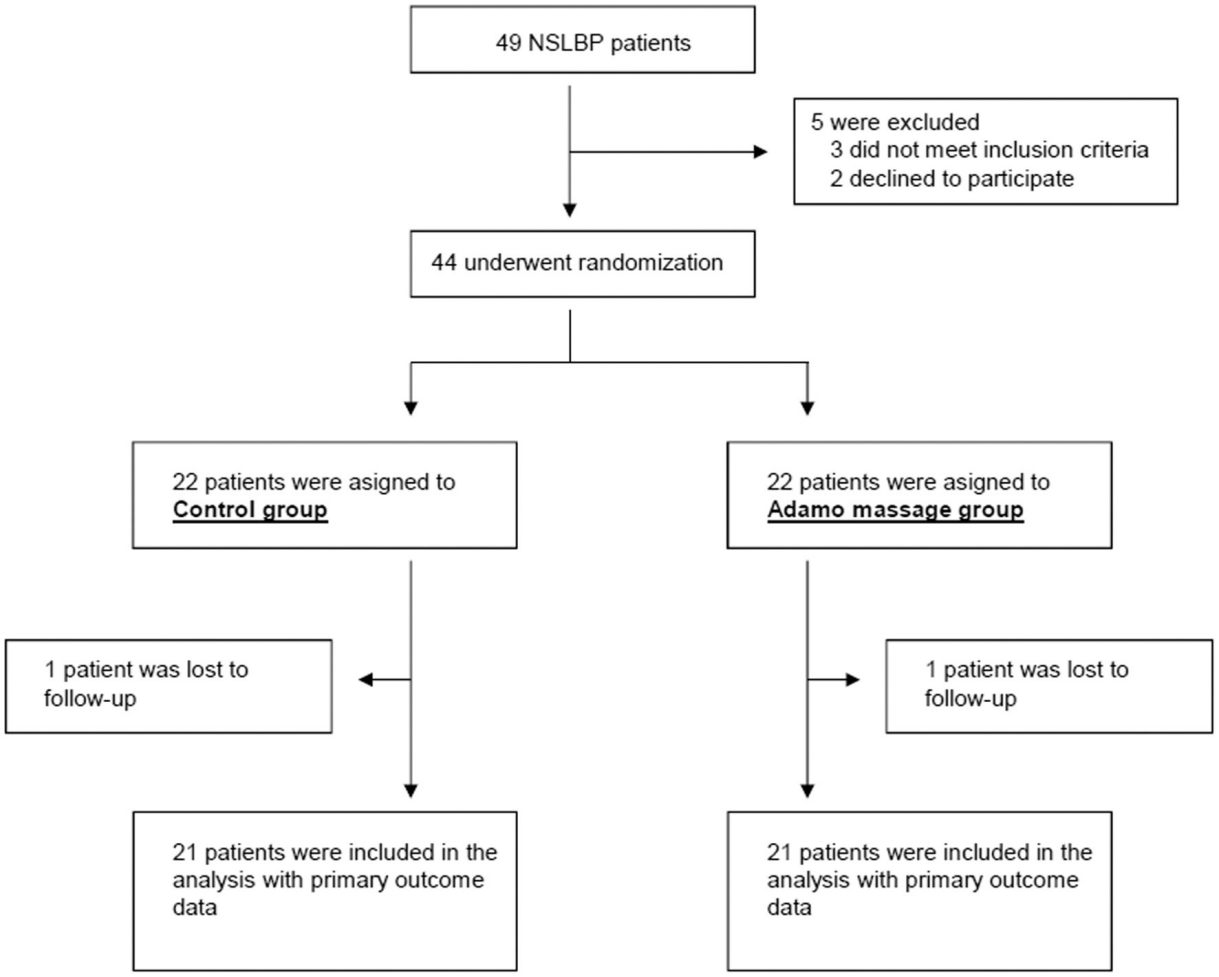
Consort flow diagram of participants through the study.

For each patient, the intervention period lasted for five weeks, with one-h exercise sessions twice a week (10 sessions). Before starting treatment, clinical characteristics of the patient were collected (age, sex, body mass index, previous treatments, etc). Then, patients were assessed with an ODI questionnaire and were randomly allocated to one of the two arms of the study: robot and control (Figure 2). Allocation was achieved with the help of an online resource that provides randomized lists (https://pinetools.com/es/aleatorizar-lista).

In the robot arm, a physiotherapist with more than 15 years of experience identified the trigger points in the patient, programmed the robot, and applied robot-controlled air pressure massage for 10 min. The ADAMO robot applies an air current to the trigger points on the back of the patient, guided by cameras and computer programs (https://adamorobot.com/) (Figure 1). Then, thermotherapy and rehabilitation exercises were provided, as is the standard treatment for NSLBP at the Rehabilitation Service (High Resolution Center San Millán).

In the control arm, patients were laid down on the robot platform. Physiotherapists identified the trigger points and the robot was connected, providing the expected background noise and vibration, but the air pressure was not applied. Thermotherapy and rehabilitation exercises were also applied.

At the end of each session, the physiotherapist applied the VAS questionnaire (not blinded). At the end of the 10 sessions, the patient went back to the physician's office to complete the second and final ODI questionnaire (the physician was blinded to the treatment).

### Outcome Measures

The primary outcome for this study was pain-related disability as tested by the Oswestry disability index (ODI). This is a questionnaire which gives a subjective percentage score of level of function (disability) in activities of daily living in those rehabilitating from low back pain. Possible scores go from 0 to 50, being 0 no pain and 50 the highest possible pain (Mehra et al., 2008). The secondary outcome was perceived pain as assessed by the visual analogue scale (VAS), which is a unidimensional measure of pain intensity. The patients are presented with a horizontal line of face pictograms. The patients mark on the line the point that they feel represents their perception of their current state, which may rank from 0 (best, no pain) to 10 (worst pain) (Haefeli and Elfering, 2006).

### Data Analysis

Categorical variables were compared using chi-square test. Normal distribution of all datasets was determined by the Shapiro-Wilk test. Since none of the datasets followed a normal distribution, only non-parametric tests were used. The temporal variation in VAS was analyzed with the Kruskal-Wallis test, followed by the Dunns *post-hoc* test. The variation on ODI and VAS questionnaires was compared between the two experimental groups with the Mann-Whitney's *U* test. *p* values lower than 0.05 were considered statistically significant. All these analyses were carried out with SPSS 17.0.

## Results

Forty-nine potential participants were approached and asked to participate in the study. Of these, three were excluded for not meeting inclusion criteria (age > 60) and two other candidates declined to participate. At the end, 44 patients were randomized into the two arms of the study, and one patient from each arm was lost during the trial. Finally, we obtained complete data from 21 patients in each arm (Figure 2).

Of the 42 patients, 14 were men and 28 women with a median age of 52 years and a median body mass index (BMI) of 27 kg/m^2^. At the beginning of the trial, they presented a median score of 15 in the ODI and 6 in the VAS. After allocation into the two arms of the study, the baseline characteristics of both populations were similar (Table 1).

**Table 1:** Clinical characteristics of patients.

	**Total**	**Control arm**	**Robot arm**	*p* value^*^
n	42	21	21	1.0^χ^
Sex, female (%)	28 (66.6%)	12 (57.1%)	16 (76.2%)	0.29^χ^
Age (years)	52 (46.75;57)	48.5 (46;57)	54 (51;58.25)	0.063^†^
BMI (kg/m^2^)	27.0 (23.7–29.5)	27.3 (24.5–32.3)	26.1 (22.7–28.1)	0.14^†^
ODI baseline	15 (10–20)	15 (9–19)	15 (11–20.5)	0.34^†^
ODI post-treatment	10 (6–15)	10 (6–15)	11 (5.5–15.5)	0.58^†^
VAS baseline	6 (4–6.5)	5 (4–6)	6 (6–7.5)	0.15^†^
VAS post-treatment	4 (2–6)	3.6 (2–6)	3.6 (2–4)	0.94^†^

*BMI, body mass index; ODI, oswestry disability index; VAS, visual analogue scale*.

*Continuous variables are presented as median and interquartile interval*.

* *Tests used in each case:*

χ *Chi square;*

† *Mann-Whitney's U test*.

**Figure 3:**
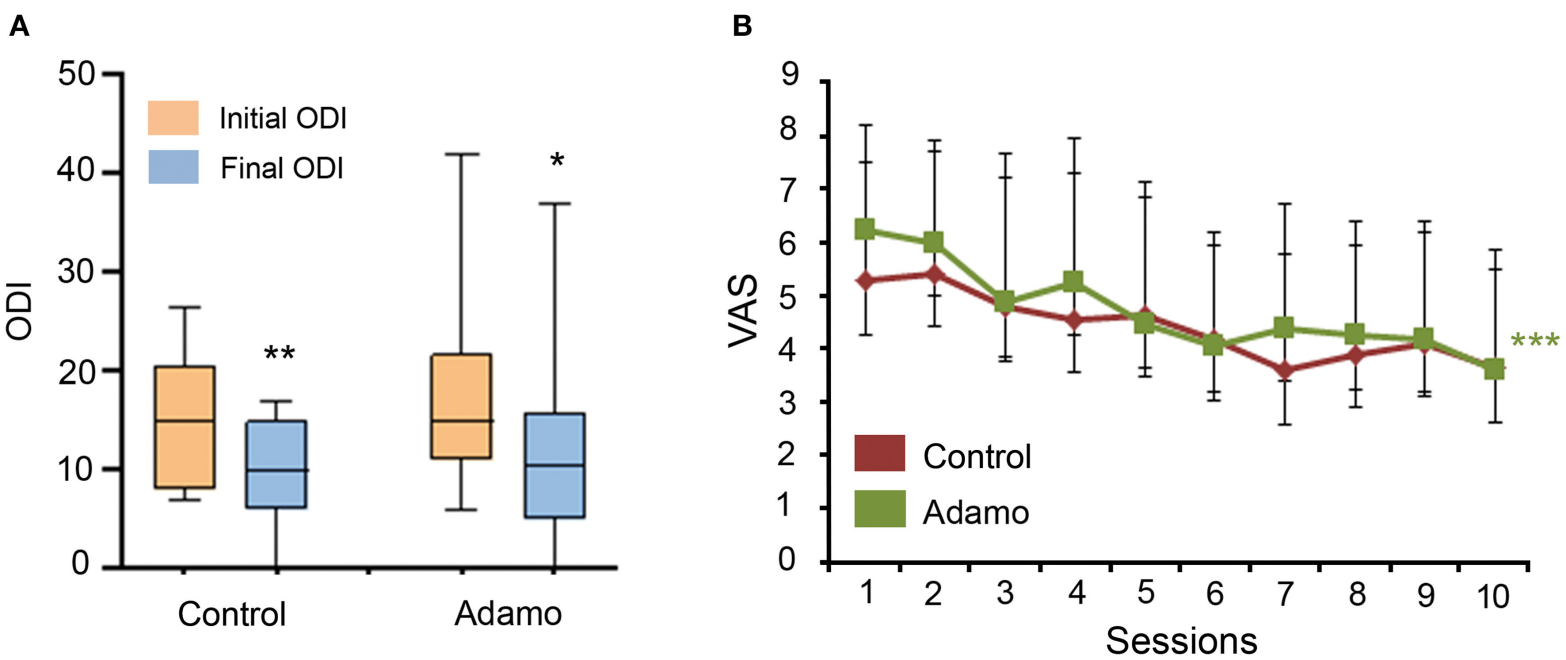
Comparison of the ODI **(A)** and VAS **(B)** scales before and after the treatment in both arms of the study. ODI values are expressed as box plots, which represent the interquartile range with the median as a horizontal line. Whiskers encompass the maximum and minimum values of the population. *p < 0.05, **p < 0.01, compared to initial ODI. VAS values are represented as the median (Q1–Q3) for each time point. ***p < 0.001, compared to initial VAS.

After 10 sessions, patients filled out another ODI questionnaire. All patients experienced a significant relief in the disability index in both arms of the trial (Figure 3A). In the control treatment, patients went from 15 (9–19) to 10 (6–15) (*p* = 0.009). In the robot treatment group, patients went from 15 (11–20.5) to 11 (5.5–15.5) (*p* = 0.036). No significant differences were found between the groups in their final ODI (*p* = 0.58). When analyzing pain perception through the VAS questionnaire, patients in the control treatment arm did not express a significant relief of their symptoms (*p* = 0.13). On the other hand, patients treated by the robot experienced a significant relief after their 10th session (*p* = 0.0001), going from an initial VAS of 6 (6–7.5) to 3.6 (2–4). There was no significant difference in VAS final values between both treatments (*p* = 0.94) (Figure 3B).

**Figure 4:**
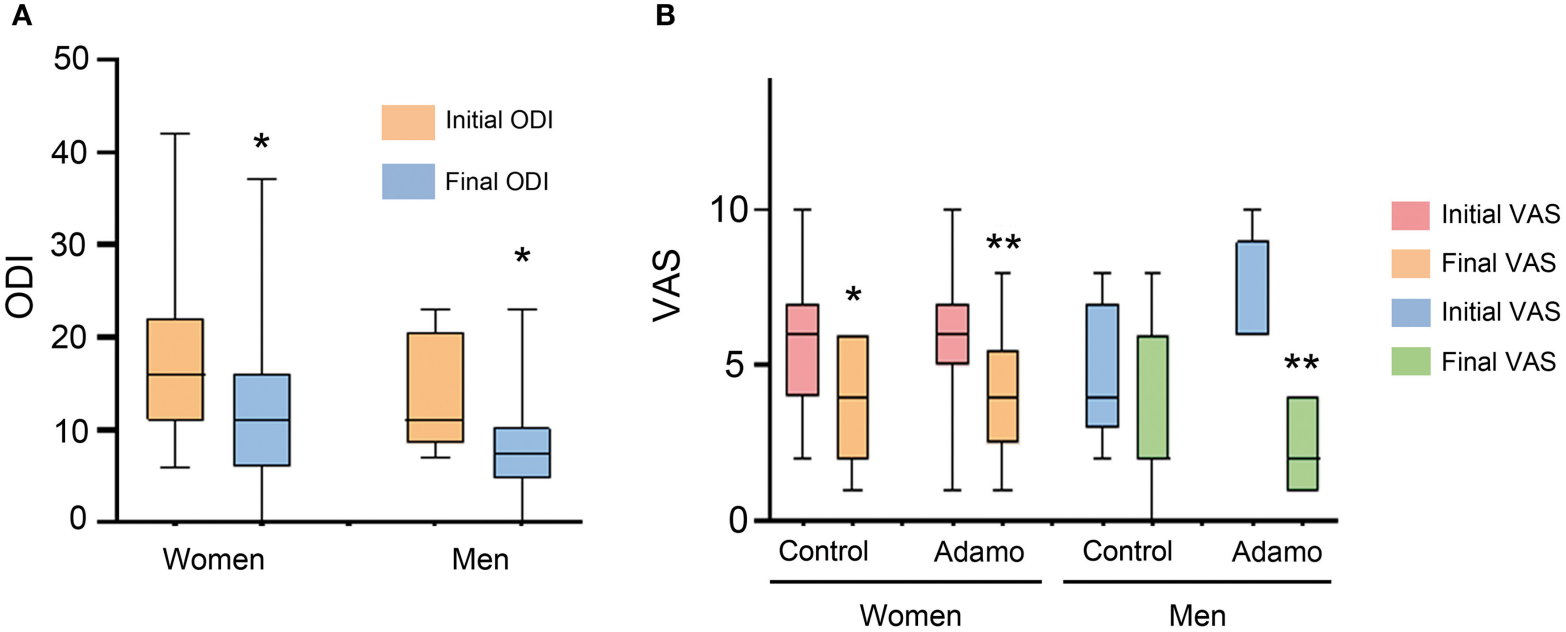
Comparison of the ODI **(A)** and VAS **(B)** scales, before and after the treatment in both arms of the study, taking into consideration the sex of the patients. Values are expressed as box plots, which represent the interquartile range with the median as a horizontal line. Whiskers encompass the maximum and minimum values of the population. *p < 0.05, **p < 0.01, compared to initial test.

Then, we investigated whether the sex of the patients had any influence in their treatment. For the ODI values, both women and men experienced a significant amelioration of their symptoms due to the treatment (Figure 4A). Regarding the VAS, women reported a significant relief for their symptoms both in the control (*p* = 0.038) and in the robot (*p* = 0.005) arms. Among men, the control treatment did not reach statistical significance (*p* = 0.36) but men treated with the robot experienced a very significant relief (*p* = 0.008), going from 6 (6–8) to 2 (1–4) (Figure 4B).

**Figure 5:**
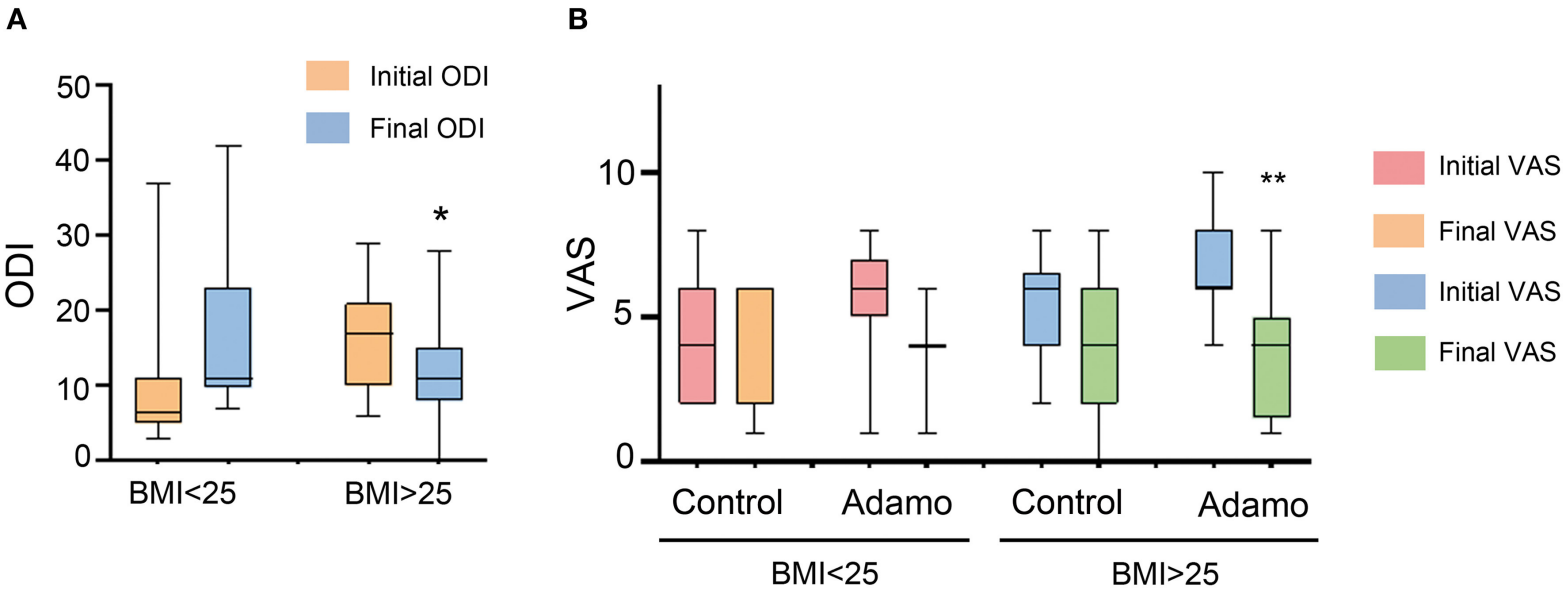
Comparison of the ODI **(A)** and VAS **(B)** scales, before and after the treatment in both arms of the study, taking into consideration the BMI of the patients. Values are expressed as box plots, which represent the interquartile range with the median as a horizontal line. Whiskers encompass the maximum and minimum values of the population. *p < 0.05, **p < 0.01, compared to initial test.

Another variable we wanted to measure was the influence of the patient's body composition, as measured by the body mass index (BMI). According to metabolic guidelines (Kahan and Manson, 2019), patients with BMI ≥ 25 kg/m^2^ are considered overweight. Clinical characteristics of these patients are summarized in Table 2. Interestingly, the best results were obtained among overweight people, both with the ODI (Figure 5A) and the VAS (Figure 5B) indexes. When analyzing the VAS data, only overweight patients treated with the robot experienced a significant relief of their symptoms (*p* = 0.001), going from 6 (6–8) to 4 (2–5).

**Table 2:** Clinical characteristics of patients divided by BMI.

	**BMI<25**			**BMI≥25**		
	**Total**	**Control arm**	**Robot arm**	**Total**	**Control arm**	**Robot arm**
n	15	7	8	27	14	13
Sex, female (%)	10 (66%)	3 (42.8%)	7 (87.5%)	18 (66.6%)	9 (64.2%)	9 (69.2%)
Age (years)	51 (47–58)	51 (48–53.7)	50 (43.7–53.7)	54 (47–57)	48.5 (46–57)	54.5 (50–59.5)
ODI baseline	11 (10–25)	10 (8–25)	11 (11–21.6)	17 (10.5–20.5)	16.5 (10.5–18.7)	20 (13–21)
ODI post-treatment	6.5 (5–10.75)	7 (5–8.5)	5 (5–13.5)	11 (8–15)	12 (8–15)	11 (9–15.5)
VAS baseline	6 (4–6.5)	4 (2–6)	6 (5–7)	6 (4–7)	6 (4–6.5)	6 (6–7.5)
VAS post-treatment	4 (2–6)	2 (2–6)	4 (4–4)	4 (2–6)	4 (2.5–6)	4 (2–5)

*BMI, body mass index; ODI, oswestry disability index; VAS, visual analogue scale*.

All individual participant data are available in Supplementary Material.

## Discussion

The aim of this randomized controlled trial was to evaluate the effectiveness of adding a robot-mediated massage to the usual treatment to alleviate NSLBP, consisting of thermotherapy and rehabilitation exercises. Both treatments were significantly beneficial for the patients and had similar effects for the primary outcome (ODI), reducing disability symptoms. On the other hand, only the robot was able to reduce perceived pain, as measured by the VAS. Interestingly, overweight patients experienced more significant relief than patients of normal weight, and this difference was more striking in the patients treated by the robot.

Obesity is a leading preventable cause of death and disease worldwide. The prevalence of obesity was 42.4% in the US in 2018. Obesity-related conditions include heart disease, stroke, type 2 diabetes, and certain types of cancer (CDC, 2021). The medical costs for people who have obesity was US$ 1,429 higher than those of normal weight (Finkelstein et al., 2009). In addition, the excessive weight generates undue stress to the joints and musculoskeletal system (Viester et al., 2013), making these people more prone to seek physical therapy treatments. Furthermore, overweight patients pose additional problems to physiotherapists that may not reach properly their trigger points during manual treatment. Our finding that robotic massage is more efficient than control treatment for overweight patients may indicate that the constant air pressure provided by the robot adds more relief to these patients.

The use of robotic devices to perform therapeutic massages has been previously reported (Wang et al., 2018; Li et al., 2020). The main difference of the ADAMO robot with other versions is that the massage is produced by a directed current of compressed air with constant intensity. This avoids direct contact with the skin, which reduces potential cross-contamination among patients and simplifies decontamination procedures. Another difference is that previous devices are based in multipurpose robotic arms that have been programmed to perform massages, whereas the ADAMO robot has been specifically designed for this single purpose.

The mechanism by which pressure and massage can diminish pain has been described. Cutaneous pressure receptors are located in the deep layers of the skin and are, mainly, the Ruffini and Pacini corpuscles (Munger and Ide, 1988). Both are connected to thick Aβ nerve fibers, which are those with the highest conduction velocity. These nerve fibers, for the most part, do not stablish synapses in the posterior horn of the spinal cord but continue to higher structures. Nevertheless, these fibers emit collateral branches toward the posterior horn where they contact pain inhibitory interneurons. Taking into account the different conduction velocity of the pain-carrying fibers (fibers C and Aδ, very slow) and of the fibers activated by pressure (Aβ, very fast), the latter produce an activation of the inhibitory interneurons and block the transmission of the nociceptive stimulus to the higher nervous centers (Garcia et al., 2021). This is in agreement with the gate control theory, which proposes that the nociceptive sensory information transmitted to the brain relies on an interplay between the inputs from nociceptive and non-nociceptive primary afferent fibers. Both inputs are normally under strong inhibitory control in the spinal cord. Under healthy conditions, pre-synaptic inhibition activated by non-nociceptive fibers modulates the afferent input from nociceptive fibers onto spinal cord neurons, while postsynaptic inhibition controls the excitability of dorsal horn neurons, and silences the non-nociceptive information flow to nociceptive-specific projection neurons (Guo and Hu, 2014). However, in addition to this mechanism, it is likely that pressure on the skin may block the release of algogenic substances (substance P, bradykinin, histamine) through resident skin cells (Schmelz, 2011). On the other hand, it cannot be ruled out that the pressure stimulus acts on the release of neurotransmitters related to pain in the posterior horn of the spinal cord (Yam et al., 2018) and on blocking the activation of the microglia responsible for central algetic sensitization and neuropathic pain (Chen et al., 2018). The pressure elicited by the robot seems to be very efficient in activating cutaneous receptors and fast fibers. Future studies must demonstrate whether the use of the robot influences the release of algogenic substances.

Addition of robot massage has shown a significant improvement in the treatment of specific patients, as well as a broader feeling of well-being (VAS score) in all patients. Nevertheless, larger effects could be expected from this technology. First, in this study, the air pressure that was applied by the robot arm was always constant. Perhaps patients would benefit from different pressures depending on their specific pathology or body type. Also, a single patient may receive different pressures in specific trigger points, depending on their thickness. Second, in this study, a trained therapist identified the trigger points. We are working on a routine that would allow the robot to identify the trigger points by itself, thus liberating a lot of extra time for the therapist. Of course, the efficiency of the robot in locating the trigger points will need to be validated with a clinical trial.

There is evidence that, despite receiving proper training in therapy postures and self-care, a high proportion of massage therapists suffer from upper extremity pain and discomfort as a result of delivering therapy treatments. The most affected areas are the wrist and the thumb, followed by the low back, neck, and shoulders (Albert et al., 2008). As expected, the intensity of the pain and discomfort experienced by the therapist is directly related with the number of patients per day and the intensity of the massage (Vieira et al., 2016). Therapeutic robots may be very useful in reducing the most damaging aspects of physical therapy, since they may substitute the therapist in performing the actual massage. Furthermore, a single therapist may coordinate several robots simultaneously, thus increasing the number of treated patients and reducing physical therapy waiting lists.

In conclusion, we have shown that the addition of massage performed by the ADAMO robot to regular non-pharmacological therapeutic protocols is at least as efficient as the control treatment, while demonstrating more efficiency in the treatment of specific patients, such as those with excessive weight. The use of massaging robots may increase the reporting and reproducibility of physical therapy protocols among different hospitals.

## Data Availability Statement

The original contributions presented in the study are included in the article/Supplementary Material, further inquiries can be directed to the corresponding author.

## Ethics Statement

The studies involving human participants were reviewed and approved by Comité de Ética de Investigación con Medicamentos de La Rioja. The patients/participants provided their written informed consent to participate in this study.

## Author Contributions

HM-M, EM, and AM: design of the study. HM-M, PM-N, SJ-M, II-G, and MR-M: patient treatment and data gathering. MB, EM, and AM: data analysis. EM and AM: funding. AM: manuscript writing. All authors approved the final version of the manuscript.

## Funding

This study was supported by a grant from Agencia de Desarrollo Económico de La Rioja (ADER), grant number: 2018-I-IDD-00039.

## Conflict of Interest

The authors declare that the research was conducted in the absence of any commercial or financial relationships that could be construed as a potential conflict of interest.

## Publisher's Note

All claims expressed in this article are solely those of the authors and do not necessarily represent those of their affiliated organizations, or those of the publisher, the editors and the reviewers. Any product that may be evaluated in this article, or claim that may be made by its manufacturer, is not guaranteed or endorsed by the publisher.
