# Supplementary material for: Using a Robot to Treat Non-specific Low Back Pain: Results From a Two-Arm, Single-Blinded, Randomized Controlled Trial
Source: Front Neurorobot. 2021 Sep 14;15:715632. doi: 10.3389/fnbot.2021.715632 (PMC8478348; doi:10.3389/fnbot.2021.715632)
Supplement: Supplementary file 1 [file Data_Sheet_1.docx]

Supplementary Material

# Individual Participant Data

**Manual arm**

| **Patient ID** | **Age** | **Sex**^†^ | **BMI** | **Initial ODI** | **Final ODI** |
| --- | --- | --- | --- | --- | --- |
| **2** | 52 | 1 | 27.2 | 11 | 14 |
| **5** | 50 | 1 | 35 | 7 | 0 |
| **7** | 46 | 0 | 39.1 | 22 | 16 |
| **10** | 57 | 0 | 32.3 | 7 | 4 |
| **11** | 47 | 0 |  |  |  |
| **14** | 58 | 0 | 27 | 15 | 15 |
| **15** | 31 | 0 | 31.1 | 18 | 15 |
| **17** | 45 | 0 | 28.2 | 16 | 10 |
| **19** | 59 | 1 | 24.5 | 11 | 10 |
| **20** | 48 | 1 | 29.2 | 17 | 6 |
| **21** | 31 | 0 | 38.2 | 10 | 8 |
| **23** | 25 | 0 | 28.58 | 19 | 15 |
| **24** | 54 | 1 | 26.47 | 23 | 10 |
| **26** | 57 | 0 | 24.34 | 26 | 7 |
| **27** | 46 | 0 | 23.44 | 7 | 4 |
| **28** | 51 | 0 | 22.68 | 26.6 | 11 |
| **32** | 48 | 1 | 24.62 | 9 | 3 |
| **33** | 49 | 0 | 32.42 | 18 | 17 |
| **37** | 60 | 1 | 21.61 | 10 | 6 |
| **40** | 48 | 1 | 22.9 | 7 | 7 |
| **41** | 45 | 1 | 32.7 | 7 | 8 |
| **43** | 57 | 0 | 27.34 | 22 | 17 |

^†^Sex: 0: Women; 1: Men.

BMI: Body mass index. ODI: Oswestry disability index.

| **Patient ID** | **VAS1** | **VAS2** | **VAS3** | **VAS4** | **VAS5** | **VAS6** | **VAS7** | **VAS8** | **VAS9** | **VAS10** |
| --- | --- | --- | --- | --- | --- | --- | --- | --- | --- | --- |
| **2** | 2 | 6 | 4 | 6 | 6 | 4 | 4 | 4 | 4 | 6 |
| **5** | 4 | 2 | 4 | 1 | 1 | 1 | 2 | 1 | 4 | 0 |
| **7** | 6 | 6 | 6 | 6 | 4 | 4 | 4 | 6 | 4 | 4 |
| **10** | 2 | 1 | 2 | 2 | 1 | 4 | 1 | 4 | 2 | 2 |
| **11** | 10 | 10 | 10 | 8 | 6 | 4 | 2 | 2 | 1 | 1 |
| **14** | 8 | 8 | 6 | 6 | 6 | 6 | 6 | 4 | 4 | 4 |
| **15** | 4 | 6 | 4 | 2 | 4 | 2 | 1 | 4 | 2 | 2 |
| **17** | 6 | 4 | 4 | 6 | 2 | 6 | 2 | 4 | 4 | 2 |
| **19** | 4 | 4 | 6 | 8 | 4 | 8 | 4 | 8 | 4 | 2 |
| **20** | 4 | 4 | 2 | 2 | 2 | 2 | 2 | 2 | 4 | 2 |
| **21** | 4 | 4 | 6 | 4 | 2 | 4 | 2 | 2 | 8 | 6 |
| **23** | 6 | 2 | 2 | 2 | 4 | 6 | 6 | 4 | 2 | 2 |
| **24** | 8 | 8 | 8 | 10 | 8 | 6 | 8 | 8 | 8 | 8 |
| **26** | 2 | 2 | 1 | 1 | 2 | 1 | 1 | 1 | 1 | 1 |
| **27** | 4 | 8 | 4 | 2 | 8 | 6 | 2 | 4 | 8 | 4 |
| **28** | 8 | 8 | 10 | 10 | 8 | 6 | 6 | 6 | 6 | 6 |
| **32** | 2 | 2 | 2 | 4 | 6 | 2 | 2 | 2 | 2 | 2 |
| **33** | 6 | 8 | 4 | 2 | 6 | 4 | 4 | 2 | 2 | 6 |
| **37** | 6 | 6 | 2 | 2 | 6 | 4 | 2 | 2 | 4 | 2 |
| **40** | 6 | 6 | 6 | 4 | 4 | 4 | 4 | 4 | 6 | 6 |
| **41** | 8 | 8 | 6 | 6 | 6 | 4 | 8 | 6 | 6 | 6 |
| **43** | 6 | 6 | 6 | 6 | 6 | 4 | 6 | 6 | 4 | 6 |

VAS: visual analogue scale.

**Robot arm**

| **Patient ID** | **Age** | **Sex**^†^ | **BMI** | **Initial ODI** | **Final ODI** |
| --- | --- | --- | --- | --- | --- |
| **1** | 49 | 0 | 22.6 | 42 | 37 |
| **3** | 58 | 1 | 25.6 | 14 | 11 |
| **4** | 38 | 0 | 19.5 | 15 | 5 |
| **6** | 60 | 1 | 42.8 | 22 | 4 |
| **8** | 59 | 0 | 21.5 | 22.2 | 16 |
| **9** | 54 | 0 | 25.9 | 9 | 6 |
| **12** | 59 | 1 | 29 | 23 | 23 |
| **13** | 54 | 0 | 24.7 |  |  |
| **16** | 39 | 0 | 22 | 11 | 11 |
| **18** | 52 | 0 | 26.4 | 6 | 0 |
| **22** | 54 | 0 | 32.44 | 21 | 21 |
| **25** | 55 | 0 | 27.73 | 20 | 15 |
| **29** | 51 | 1 | 23.94 | 10 | 5 |
| **30** | 51 | 1 | 29.86 | 20 | 9 |
| **31** | 46 | 0 | 28.13 | 20 | 10 |
| **34** | 59 | 0 | 29.74 | 13 | 12 |
| **35** | 57 | 0 | 27.34 | 15 | 12 |
| **36** | 57 | 0 | 28.12 | 29 | 28 |
| **38** | 54 | 0 | 27.01 | 10 | 10 |
| **39** | 52 | 0 | 21.5 |  |  |
| **42** | 45 | 0 | 23.12 | 11 | 5 |
| **44** | 62 | 0 | 22.32 | 11 | 5 |

^†^Sex: 0: Women; 1: Men.

BMI: Body mass index. ODI: Oswestry disability index.

| **Patient ID** | **VAS1** | **VAS2** | **VAS3** | **VAS4** | **VAS5** | **VAS6** | **VAS7** | **VAS8** | **VAS9** | **VAS10** |
| --- | --- | --- | --- | --- | --- | --- | --- | --- | --- | --- |
| **1** | 1 | 6 | 1 | 1 | 1 | 1 | 2 | 1 | 1 | 1 |
| **3** | 6 | 6 | 8 | 8 | 6 | 4 | 4 | 4 | 2 | 2 |
| **4** | 6 | 6 | 2 | 4 | 2 | 1 | 1 | 2 | 2 | 4 |
| **6** | 8 | 6 | 6 | 6 | 6 | 6 | 4 | 4 | 4 | 4 |
| **8** | 8 | 8 | 8 | 6 | 6 | 6 | 6 | 6 | 6 | 6 |
| **9** | 4 | 2 | 2 | 2 | 2 | 1 | 1 | 2 | 4 | 2 |
| **12** | 10 | 10 | 10 | 10 | 10 | 8 | 10 | 10 | 1 | 1 |
| **13** | 8 | 6 |  |  |  |  |  |  |  |  |
| **16** | 4 | 4 | 4 | 2 | 1 | 4 | 4 | 4 | 2 | 4 |
| **18** | 6 | 4 | 2 | 4 | 2 | 4 | 2 | 2 | 2 | 2 |
| **22** | 6 | 8 | 2 | 10 | 4 | 2 | 8 | 2 | 8 | 1 |
| **25** | 10 | 6 | 10 | 10 | 10 | 8 | 8 | 8 | 8 | 8 |
| **29** | 6 | 6 | 4 | 6 | 4 | 4 | 4 | 6 | 6 | 4 |
| **30** | 6 | 4 | 4 | 4 | 2 | 2 | 2 | 2 | 2 | 1 |
| **31** | 6 | 6 | 8 | 4 | 8 | 4 | 6 | 4 | 4 | 4 |
| **34** | 4 | 4 | 1 | 1 | 2 | 4 | 2 | 4 | 4 | 4 |
| **35** | 6 | 8 | 4 | 4 | 4 | 4 | 4 | 4 | 8 | 6 |
| **36** | 6 | 6 | 6 | 6 | 4 | 4 | 6 | 6 | 4 | 4 |
| **38** | 8 | 8 | 8 | 8 | 8 | 8 | 6 | 6 | 6 | 6 |
| **39** | 6 | 6 | 4 | 6 | 4 | 2 | 4 | 4 | 4 | 4 |
| **42** | 6 | 6 | 4 | 4 | 4 | 4 | 4 | 4 | 6 | 4 |
| **44** | 6 | 6 | 4 | 4 | 4 | 4 | 4 | 4 | 4 | 4 |

VAS: visual analogue scale.
